# From Pachymeningitis Hemorrhagica to Abusive Head Trauma: A Journey Through the Centuries

**DOI:** 10.1177/10775595251361930

**Published:** 2025-07-18

**Authors:** Oliver Berthold, Flora Blangis, Christopher Spencer Greeley

**Affiliations:** 1 Department of Child and Adolescent Psychiatry and Psychotherapy, Ulm University Hospital, Ulm, Germany; 2 Child Protection Clinic, Department of Pediatrics, DRK Kliniken Berlin, Germany; 3 Obstetrical, Perinatal and Pediatric Epidemiology Research Team, Centre of Research in Epidemiology and StatisticS, Université Paris Cité, 27102 INSERM, Paris, France; 4 Inserm CIC 1413, Nantes University Hospital, Nantes, France; 5 Child Protection Unit-Department of General Pediatrics and Pediatric Infectious Diseases, AP-HP, Necker-Enfants Malades Hospital, Université Paris Cité, Paris, France; 6 Division of Public Health Pediatrics, Texas Children’s Hospital, Baylor College of Medicine, Houston, TX, USA

**Keywords:** abusive head trauma, child abuse, shaken baby syndrome, medical history

## Abstract

The development of medical knowledge follows specific principles. These also apply to abusive head trauma. The seminal literature from the early 20^th^ century is based on earlier research from Europe, which is fundamental to understand the evolution of understanding and nomenclature of abusive head trauma. A review of original medical literature on intracranial injuries in children, published in German, English, and French, was conducted using PubMed and Google Scholar for articles before 1950. The primary search focused on subdural hemorrhages and pachymeningitis in children, followed by a snowball search of references. Full texts of available manuscripts were reviewed. Primary literature as early as 1839 was reviewed by native language speakers, referring to intracranial collections of blood that underwent an evolution in nomenclature and understanding of causation. Initially termed *pachymeningitis hemorrhagica*, implying an inflammatory cause, the nomenclature evolved to subdural hematoma, as traumatic causes aligned with clinical experience. Advances in diagnostic imaging further enhanced understanding and nomenclature. The clinical findings associated with abusive head trauma have been described for centuries, with consistent signs and symptoms until the present day. As the understanding of the disease evolved due to modern diagnostic techniques, changes in nomenclature became necessary.

## Introduction

The march of science is the embodiment of change. Human knowledge continues to evolve, and as human knowledge evolves the understanding of our experiential world matures. Throughout the 19^th^ and 20^th^ centuries, how we accumulate knowledge has been the subject of much scholarship. Although being traced back to Socrates and Plato, modern epistemology starts in the early 19^th^ Century with Hegel’s *Dialectic* (Hegel, 1807). This is the description of how knowledge evolves from resolution of conflict between two opposing ideas (“thesis”, “antithesis”). The resolution of this conflict results in the “synthesis” (of knowledge). This cyclic evolution of knowledge has been modernized by Thomas Kuhn in *The Structure of Scientific Revolutions* (Kuhn, 1970). This iconic text is credited with coining the contemporary usage of the term “paradigm” to describe large frameworks for understanding. These paradigms are repeatedly tested and challenged until enough new data is accumulated, resulting in a “paradigm shift”. Often, the term “scientific process” is used to frame how understanding can be this march of science and how knowledge undergoes standard proceeding to result in reliable outputs. Karl Popper is best known for *The Logic Of Scientific Discovery*, in which he proposes “falsifiability” and “verifiability” as the underpinnings of a clearly articulated methodology of discovery (Popper, 2002). Where “falsifiability” is a structured scientific principle that could, in theory, be falsified. A “non-falsifiable” theory should be considered pseudoscience. As applied to clinical research and scientific discovery, Chalmers describes a more recognizable framing (Chalmers, 2013). Knowledge begins with hypothesis generation (similar to Popper), which then requires the scientist to collect facts (data) via observation. Repeated observation is the process of inductive reasoning”. Induction results in the development and refinement of “laws” and principles” regarding the proposed hypothesis. At this point, putative “laws and principles” can be tested through “deduction” in which a theory is attempted to be falsified (i.e. testing the Null Hypothesis).

Medicine itself is an evolving branch of science. Many diseases have been known for centuries without a clear understanding of the underlying cause, some even being attributed to a mythological source, that is the Black Death. Other conditions known today to be signs or symptoms of one particular disease were thought to represent completely different diseases, that is scurvy. Theories of causation and treatment had to evolve before modern concepts and diagnostic strategies made effective prevention and treatment possible. “Laws” and theories” are developed and refined, and deductive exploration can help uncover potential causes. Proving a causal relationship often requires significant scientific, theoretical or analytic effort beyond simple observation (Hill, 2016). In 1965, Hill published nine principles that help to establish, whether a certain exposure is the cause of a condition when direct experiments are not possible (e.g. for ethical reasons) (Hill, 1965).

This article is about medical history rather than about medical knowledge. However, a basic understanding of how medical knowledge develops and how the quality of theories can be assessed is helpful (Chalmers, 2013). As described above by Chalmers, clinical observation (induction) often only allows theories about the relationship between cause and effect to be proposed initially (hypothesis generating”). In this article, we outlined the history of abusive head trauma (AHT) as it has been reported in the published literature, and particularly how the understanding of causation of its signs and symptoms have evolved.

Because some of the old misconceptions and long-debunked flaws occasionally resurface as old wine in new bottles, it is important to look back to history in an attempt to avoid repeating mistakes. It is therefore inevitable that we begin our historical journey with subdural hemorrhages. This condition has been known to exist for almost two centuries and has been regularly linked to a history of trauma (Baillarger, 1839; Tardieu, 1868). However, in cases without a history of trauma, its origin has puzzled clinicians and pathologists alike, leading to a variety of assumptions and theories about the phenomenon. We used original historical sources in English, German, and French to trace observations stemming from the 19^th^ century to the 20^th^ century. Application of theories of causation to child physical abuse and AHT in particular will be part II of this article series.

In an earlier article by Binney, the history of AHT has already been examined from a perspective of epistemology (Binney, 2023). However, the author makes various assumptions which are also found in articles that question the existence of the condition per se. This includes equating the broad international scientific consensus on AHT with mostly narrative publications that question this concept. The article purports to assess historical literature but is limited to the latter half of the 20^th^ century, restricted to publications from the U.S., with the well-known articles by Caffey and Guthkelch. Consequently, the article ignores the broad basis of earlier scholarly literature that Caffey and Guthkelch had been built upon. Moreover, the amalgamation of medical and legal concepts is a prevalent error in the interpretation of the diagnosis of AHT. The requirement that something be proven “beyond reasonable doubt” pertains to the individual guilt of a defendant in court and is not a scientific (or even an epistemological) framing. This is a specious standard, as it does not apply to a single piece of evidence, let alone to a medical diagnosis. Consequently, the article’s conclusion that the epistemological basis of AHT did not withstand close examination is founded on insufficient data and erroneous assumptions.

The purpose of this manuscript was to synthesize the evolution of the understanding of head injuries in infants by utilizing the original sources in their original language. Our hypothesis was that head injuries in infants would be described in the historical literature, but perhaps in not terms or understanding as is contemporary. This would be in keeping with how scientific and medical understanding is an iterative process, continually being updated and refined.

## Methods

We conducted a non-systematic scoping review on historical medical literature about intracranial collections in children published in German, English, and French searching Medline via PubMed and Google Scholar for literature before 1950 (i.e., before Caffey’s first article on long bone fractures and chronic subdural hematoma was published (Caffey, 1946)). The primary search included terms for injury as well as potential findings (i.e., subdural, pachymeningitis, fracture, hematoma, bleeding) in children. A secondary snowball search of all references from all retrieved studies was performed until no further studies were identified. Articles were included if they included subdural collections, pachymeningitis, bruising or fractures in children. Full texts of all retrieved manuscripts were reviewed, if available. Articles in English language were assessed by CSG, articles in German language by OB, and articles in French by FB.

## Results

### A Short (Modern) History of Head Injuries in Infants

AHT has been defined by a 2008 Centers for Disease Control and Prevention’s expert panel and this definition was affirmed by the recent Academy of Pediatrics’ technical report: “an injury to the skull or intracranial contents of an infant or young child (<5 years of age) due to inflicted blunt impact and/or violent shaking” (Narang et al., 2025; Parks et al., 2012).

The entity has been known for centuries, probably millennia (Nystrom, 2020; Wheeler et al., 2013). In the 17^th^ century, Paolo Zacchia, who was personal physician to Popes Innocentius X and Alexander VII, described the infliction of head trauma on a child by a teacher (Labbe, 2005). Here, we have to rely on secondary literature because it was not possible for us to find and evaluate the original source in Latin.

James Parkinson, the British physician who was the first to describe the „shaking palsy“, delicately warned parents in 1812 that excessive force to the head might cause hydrocephalus: *“Parents must excuse the suggestion, as it is made with a hope that it may prove beneficial. – The correction of children, performed in the moment of passion, is not always within those bounds which the parent would the moment before, or after the infliction of the punishment, himself have prescribed. A box on the ear, as it is termed, or a severe blow on the head with the open hand, is the most ready punishment, and therefore most generally adopted, when petulance or passion impels to an immediate correction. But when I consider the tender fabric of the brain, and also that a blow sufficient to give the intended degree of pain to the delinquent cannot be inflicted without giving a considerable jar to the head, I must suspect it to be a mode of correction highly improper, and which may even possibly occasion this dreadful malady.”* (Parkinson, 1812, p. 419). Two things are particularly remarkable about Parkinson’s comments: First, he described the infliction of a severe head injury to a child by the violent act of a parent, which can lead to hydrocephalus (through subdural hemorrhage and hygroma) – almost every aspect that constitutes the modern definition of AHT. Second, the cautious manner in which Parkinson put his warning. Parkinson induced (observed) a constellation of findings and subsequently deduced (hypothesized) a potential explanatory theory for his findings. This follows the normal procedure of the scientific process.

James Parkinson focused on intracranial injury (“watery head” in his words, hydrocephalus in ours). Hydrocephalus, a collection of fluid in the intracranial space might be caused by an obstruction of cerebrospinal fluid circulation or secondary to subdural hemorrhages from traumatic causes, including AHT (Wittschieber et al., 2015). Our search of 19^th^-century literature from the United Kingdom revealed no other references to subdural hematomas in children. Instead, it is noteworthy to review the literature of the late 19^th^ century in German-speaking countries and France, where two conflicting theories about the causes of subdural collections were prevalent.

### “Pachymeningitis Haemorrhagica Interna”: A Common, but Unexplained Condition in the 19^th^ Century in Continental Europe

In French literature of the 19^th^ century, subdural collections were mostly considered sequelae of subdural hemorrhage regardless of the cause (Baillarger, 1839), while in 1856, Rudolf Virchow coined the term “pachymeningitis interna haemorrhagica” for what French pathologists had previously described as subdural collections of blood (Virchow, 1857). Virchow claimed that the membranes he found in these cases were of inflammatory origin rather than organized hematoma. Therefore, he called the finding “pachymeningitis”, referring to the assumed infection.

Virchow enjoyed a huge influence on the advancement of medical science in the second half of the 19^th^ century. Therefore, his theory of the causes of pachymeningitis caused a detour in the interpretation of the findings in the German-speaking medical community, as subsequently clinicians tried to identify a causative infectious agent for the condition.

In France, during the same period, Ambroise Tardieu, who is often referred to as the pioneer of child abuse medicine (Labbe, 2005; Roche et al., 2005), published in 1860 a report of 32 children with abusive injuries that was groundbreaking then and remains of relevance to this day (Tardieu, 1860). In 24 of his 32 cases, he was clearly able to attribute the perpetration of the trauma to the parents (Tardieu, 1860). Having a clear history of trauma, for him there was no doubt that “numerous bruises on the head and limbs, and on the surface of the brain an effusion of blood result(ed) from the blows inflicted” (translated by FB from the original source). In another case report, he described the case of a three-year-old child, having been seized violently by his father and thrown on the head. “After the fall, the child vomited (…) suffered delirium, fever, vomiting and then coma for three days. (…) There are no external marks (…), but inside we find the characteristic lesions of contusions of the brain and spinal cord, bruising of the brain, red spots, softening (…). The illness and death cannot be considered accidental and are the direct result of the violence to which the child was subjected three days before his death.” (translated by FB from the original source).

For Tardieu, in his case reports the link between intracranial findings and violence in children was obvious simply because he performed autopsies on children who died from violent assaults. In Germany, extensive empirical data is available from this period where autopsies were performed on children who had died from *unknown *causes. For decades, German scholars therefore did not establish the link that was obvious to Tardieu.

In 1890, forensic pathologist Doehle of Kiel in Germany published autopsy findings of 38 deceased infants with “pachymeningitis”. Many of those infants were described as “atrophic”, that is malnourished, so that the suspicion of neglect was raised in many cases. However, Doehle hypothesized that in these cases traumatic bleeding due to birth trauma had caused brain damage and subsequent failure to thrive (Doehle, 1891). This theory was challenged by contemporary colleagues as the infants were usually considered too old to show signs of birth trauma. One case report by Doehle is particularly noteworthy from today’s point of view: An infant allegedly fell from the bed to the floor and was found dead by the arriving physician. In autopsy, facial bruising and skin abrasions, occipital swelling and subdural bleeding were noted. As the infant had been sick with bronchitis before his death, Doehle hypothesized that coughing might have led to subdural hemorrhage and motor dysfunction, subsequently causing the fall (Doehle, 1891). Two years later, in 1892, forensic pathologist Lesser wrote: “It (pachymeningitis interna haemorrhagica) can be the consequence of habitual abuse by cruel parents or caregivers towards young subjects. Four such cases I dissected myself.” (translation from German by OB) (Lesser, 1892).

While German forensic physicians and pathologists demonstrated a stronger tendency to associate pachymeningitis with traumatic causes, clinicians, particularly from the then young subspecialty of pediatrics, still tried to find the infectious cause postulated by Virchow about half a century earlier.

### 1900-1920: Retinal Hemorrhages Gain Attention as a Common Co-Occurrence of Subdural Collections

At the turn of the century, the condition of “pachymeningitis interna haemorrhagica” was a common topic in pediatric publications and on congresses in German-speaking literature, demonstrating that the condition was not only an unsolved problem but also a common finding for the clinical pediatricians, who at that time usually performed autopsies on their own patients (Finkelstein, 1904; Göppert, 1905; Hahn, 1911; Misch, 1905; Rosenberg, 1913). A group of pediatricians who worked in the municipal orphanage of Berlin took a leading role in advancing the understanding of the condition. In 1904, Heinrich Finkelstein reported the case of a 7-month-old infant from the orphanage. The child had lived in a foster family but was “returned” due to an increased growth of the head circumference. The child showed signs of spastic hemiplegia in the lower limbs, hydrocephalus, and extensive both cloudy and streaky retinal hemorrhages. Lumbar puncture yielded clear liquid at high pressure and puncture through the fontanel yielded fresh blood. He made the diagnosis of “pachymeningitis interna haemorrhagica”, even though a causative infection could not be found (Finkelstein, 1904).

In 1905, Peter Misch, one of Finkelstein’s residents, published two cases in infants with pachymeningitis haemorrhagica, retinal hemorrhages, and papilledema of “unknown etiology” (Misch, 1905).

For Finkelstein, the two conflicting theories of causation by Virchow and French scholars were in fact two distinct conditions. One “proper”, progressive pachymeningitis, accompanied by a progressive course of hydrocephalus and spasticity, due to infection and the other, stationary form, often due to trauma, usually birth trauma. Both forms, according to Finkelstein, were characterized by retinal hemorrhages (Finkelstein, 1904, 1905; Misch, 1905).

It is worthwhile comparing the cases reported by Tardieu from 1860 and those of Finkelstein, Misch and Rosenberg, published more than 40 years later. All describe patients, who often had a deprived and malnourished appearance, were reported to be “pale” and “thin” (Labbe, 2005). The clinical findings resemble each other: Tardieu describes multiple bruises and hydrocephaly in one case of an infant, bruises on the head and limbs and a “blood collection covering the brain” in another case of a 2-year-old (Labbe, 2005). Rosenberg reports an infant with hydrocephaly, facial bruising, retinal hemorrhages, bloody cerebrospinal fluid, and another one, acutely ill appearing, with vomiting, multiple bruising and petechiae to the face and bloody cerebrospinal fluid (Rosenberg, 1921). The key difference is the history: Tardieu had clear histories of (abusive) trauma and was easily able to assign the findings to the accounts of abusing parents. The pediatricians working 40 years later had no history of trauma and were influenced by Virchow’s theory of inflammation. However, they failed to find a causing agent and were left puzzled as to what the cause of this condition might be.

The largest dataset of autopsies in children was published by Kowitz in 1914. Kowitz published the findings of 5,998 autopsies on children under the age of 2 from 1889 to 1911. He finally dismissed the theory of inflammatory origin and clearly stated that the causative agent of pachymeningitis always had been subdural hemorrhages (“Only the subdural hemorrhages come into question for the genesis of the hemorrhagic pachymeningitis”). In cases where infection was found at the time of death, it was always secondary or unconnected to the subdural hemorrhage. He was also the first to discuss a possible correlation between infanticide and pachymeningitis (Kowitz, 1914). From this time, the traumatic origin of pachymeningitis in children was undisputed among German pathologists, but not pediatricians.

Oskar Rosenberg succeeded Finkelstein as chief pediatrician in the Berlin municipal orphanage (“Städtische Kinderfürsorge”) in 1918 and continued his teacher’s pursuit for a better understanding of the often-debilitating condition. In 1913, he had reported the observation which had been made in 1890 by Doehle and repeated by the Swiss pediatrician Emil Feer in 1916: that pachymeningitis interna haemorrhagica did not affect all children equally. According to Doehle and Feer, mostly chronically malnourished children were affected (Doehle, 1891; Feer, 1916). Rosenberg observed in 1913 that pachymeningitis haemorrhagica interna was dramatically more common among children in the orphanage compared to children in private practices or hospitals (Rosenberg, 1913). He concluded that “pachymeningitis is only common in institutions and thus constitutes a particularly saddening variety of hospitalism” (translation by OB) (Rosenberg, 1921). Although in several case reports Rosenberg reported the presence of injuries today known to be sentinel injuries for AHT (mostly facial hematomas), inflicted trauma did not occur to him as a cause for pachymeningitis haemorrhagica interna. To the best of our knowledge, the frequent occurrence among children under institutionalized care in Berlin in the first two decades of the 20^th^ century has not been investigated to date.

In the early 20^th^ century, the phenomenon of pachymeningitis and retinal hemorrhages begins to reflect in the English literature as well (Schwartz, 1916).

In 1914, British neurosurgeon Wilfred Trotter noted that subdural hematoma were of venous origin because of their slow development and was the first to identify the veins that run at right angles from the surface of the brain to the dura as the vessels of origin-today known as bridging veins (Trotter, 1914). Trotter also made two important points. First, he described cases (of adults) with subdural hemorrhage and noted that the clinical courses were very similar to those described by other authors for pachymeningitis hemorrhagica interna. Therefore, he concluded that “it may be taken fairly certain that the primary condition is a haemorrhage, and that this comes from a venous source”. And second, that practically all his cases of subdural hemorrhages were of traumatic origin. “Internal haemorrhagic pachymeningitis” was a term which involved an unjustified hypothesis and should “be discarded in favour of some such term as chronic subdural haemorrhage”, and that “haemorrhagic pachymeningitis, being a purely traumatic lesion” (Trotter, 1914).

### 1920-1950: Subdural and Retinal Hemorrhages Form a Common Syndrome

Burhans and Gerstenberger (Burhans & Gerstenberger, 1923) reported on 5 infants with “internal hemorrhagic pachymeningitis in infancy” noting “[T]here has always been a controversy regarding the pathogenesis of the disease.” They reviewed the case reports and case series published up to that point and particularly noted the association of retinal hemorrhages: “The presence of retinal hemorrhages makes the diagnosis practically certain, and a positive fontanel puncture is pathognomonic… The most characteristic finding is the retinal hemorrhage, which is present in a majority of the cases and is certainly rare in other conditions of later infancy.” They also highlight residual neurologic sequelae, noting “[M]any of the infants who live through the attack carry a permanent disability, such as chronic hydrocephalus, imbecility, blindness, deafness, paralysis, spasticity or speech defect.”

In 1930, Sherwood published a comprehensive literature review of the German, French, and English articles on the subject that had appeared up to that point, followed by a series of nine cases of his own (Sherwood, 1930). In summary, he described that the etiology in adult patients was less puzzling, as trauma could often be identified. In children, infants before the age of six months were particularly affected and practically all common infections at the time were postulated as possible causes without convincing evidence. Basically, many authors of the case reports were only certain of the cause if a trauma could be identified (Sherwood, 1930). The same is true for Sherwood’s nine cases: he described “dubious home conditions”, an old fracture of the radius in one patient without a history of trauma and discussed the possibility of trauma without admission of it. Furthermore, similar to (Burhans & Gerstenberger, 1923), the most common finding that was not directly attributable to the hydrocephalus itself was retinal hemorrhages, which were present in over 50% of the cases both in the literature and in his own sample. If the patients are divided according to the acuity of the onset of symptoms, a different picture emerges: All patients who had been examined within a few days of symptom onset showed retinal hemorrhages. Remarkably, many of the children had been cared for in foster homes or state institutions.

In 1932, Peet and Kahn published a case series of 9 infants with subdural hematoma (Peet, 1932). They reported that subdural hematomas were usually referred to as idiopathic hydrocephalus and emphasize the need for differential diagnosis and similar to (Burhans & Gerstenberger, 1923), they proposed retinal hemorrhages as a diagnostic criterion to distinguish subdural hemorrhages from idiopathic hydrocephalus. Interestingly, Peet and Kahn emphasized that trauma was the main cause of this common condition in infants - but cautioned against putting this into context when no history of trauma was reported in their case reports. They still used Virchow’s old term of pachymeningitis hemorrhagica in some patients, but again without contextualizing this as a possible traumatic cause.

In the 1930s, there was a trend to refine the nomenclature from “haemorrhagic pachymeningitis” to “subdural hematoma”. In their series of more than 50 infants and children with subdural hematomas from the US, Ingraham and Heyl referred to Rosenberg as well (Ingraham & Heyl, 1939). They particularly referred to Rosenberg’s observation of social risk factors for subdural hematoma in infants. Furthermore, they were examining scurvy as a cause of subdural hematoma but concluded that although the incidence of scurvy in children has decreased significantly due to better nutrition, there was an *increasing *incidence of subdural hemorrhages (Ingraham & Heyl, 1939). They noted that infants with poor diet and subsequent Vitamin C deficiency were also at risk of poor care and injury; “(…) we realize that the illegitimate infant, or the one given poor general care, may well have a deficient diet and also be more frequently exposed to trauma than is the child living under ideal conditions (…)”. They reported cases of infants without a history of trauma, with normal Vitamin C levels, subdural hemorrhage, long bone fractures, bruising and retinal hemorrhage. They noted in the more than 50 cases they reviewed that “Trauma has been a more constant feature in the history of our cases, although it has not been present in all.” The lack of trauma history was not questioned, even though the cases with and without trauma showed clear parallels (Ingraham & Heyl, 1939).

It was only seven years later that Caffey published his widely known article on “Multiple fractures in the long bones of infants suffering from chronic subdural hematoma” (Caffey, 1946): “For many years we have been puzzled by the roentgen disclosure of fresh, healing and healed multiple fractures in the long bones of infants whose principal disease was chronic subdural hematoma” (Caffey, 1946). This development had two prerequisites: first, the evolution in understanding of subdural hemorrhage that had led to leaving behind the old concept of pachymeningitis and second, the development and the increasingly routine use of X-ray imaging.

Caffey summarized the former evolution of understanding, stating that “the traumatic theory of the causation of subdural hematoma has been accepted almost to the exclusion of all other causes despite the fact that a history of trauma is lacking (…)”. Also, for the long bone fractures in his sample, only traumatic injuries are possible, despite the lack of history, “the motive for denial has not been established” (Caffey, 1946).

## Discussion

### Historical Literature in the Context of Modern Understanding

It is remarkable how clear Parkinson was in his description of abusive head injuries and how carefully he put his works, advising parents to refrain from such “corrections” (even if this might be partly due to the language of the time). We hypothesize that he was either concerned that his warning could be perceived as an inappropriate interference in private affairs of families or that his connection between cause and consequence would not go unchallenged.

Finkelstein and his colleagues deserve the credit for having presented meticulous descriptions of key findings of AHT, particularly subdural hemorrhages, clinical signs and symptoms in combination with retinal bleedings. However, they interpreted different clinical manifestations as two distinct entities–perhaps because the time to dismiss Virchow’s theory had not yet arrived, and additionally the idea that a caregiver could inflict severe violence on an infant without reporting any kind of trauma was not yet comprehensible to them–despite Lesser’s conclusion over a decade earlier.

Sixteen years after Caffey, C. Henry Kempe published his conclusion that the reason for the lack of a history of trauma in cases of clear traumatic injuries was that it was too embarrassing for the caregivers to disclose the fact that a child had been physically assaulted. He coined the term “battered child syndrome” (Kempe et al., 1962).

A further step towards a more precise understanding of the cause and nature of subdural hemorrhages in abused children was both accounts of perpetrators who described how they had shaken an infant and the comparison of injury to those that adults suffered from severe rear to front car collisions before head rests became compulsory–the so called whiplash injuries, a term first used for abusive injuries in infants by Guthkelch in 1971 (Guthkelch, 1971) and further developed by John Caffey (Caffey, 1974).

Though this understanding was challenged by scholars who argued that children with whiplash shaken infant syndrome did not display the cervical injuries typical to adults involved in car accidents (Ommaya et al., 1968), this formulation became the dominant theory of subdural hemorrhage by the late 20^th^ century. However, modern imaging protocols involving advanced magnetic resonance technology needed to be developed to show that cervical injury in fact is common in children with AHT–reflected in the published literature from the 1990s until today (Brennan et al., 2009; Choudhary, 2020; Feldman et al., 1997; Kemp et al., 2010).

### The Missing Link

The path from Parkinson through Kempe to the international consensus statement has been quite tortured. More than 80 years of published literature has demonstrated a number of typical “scientific process” characteristics. There has been an iterative evolution from induction to deduction that is built upon prior knowledge to an increasingly mature theory of causation of AHT. Authors have proposed theories, and subsequent authors have expanded upon their theories, with reported features being refined to present a more complex and nuanced spectrum of findings. Lacking the understanding of the common cause, it was not possible for pediatricians in the 19^th^ century to assign such different findings as subdural hemorrhages, retinal hemorrhages, bruises or fractures to a common disease at their time. While trauma had been included as a potential cause beginning in the late 19th century, authors remained cautious to not over interpret their findings. It is clear that Virchow’s influence on pediatricians at the time was still strong and that they were unable to break away from his belief in the role of infection. Sherwood aptly summarizes Virchow’s overbearing role in the history of this disease: “Before Virchow’s time the condition was recognized, and trauma was given as an etiologic factor. In 1856 however, Virchow described the disease and suggested infection as a cause. Since that time much discussion has taken place in regard to the etiology” (Sherwood, 1930).

In the transition into the second half of the 20^th^ century, a considerable body of professional literature has been published on AHT, beginning with the seminal works of C. Henry Kempe and John Caffey (Caffey, 1946; Kempe et al., 1962). Thanks to Kempe, Caffey, and their medical and scientific successors, scientific societies around the world engaged in the treatment of children, provided guidelines on diagnosis, treatment, and intervention of AHT (Blesken et al., 2019; Choudhary et al., 2018; Christian et al., 2009; Shanahan et al., 2013; Sieswerda-Hoogendoorn et al., 2012; Wootton-Gorges et al., 2017). The challenge of the entity itself became reduced to an issue before courts of law as a defense argument (Greeley, 2014; Narang & Clarke, 2014).

A consensus statement by many international scientific societies, including the American Academy of Pediatrics (AAP), the American Professional Society on the Abuse of Children (APSAC), Swedish, Norwegian and Japanese pediatric societies and US-American and European societies for pediatric radiology was published in 2018 (Choudhary et al., 2018). It defined AHT as the clinical picture of intracranial and spinal injury, complex retinal hemorrhages, and rib and other fractures inflicted by shaking, shaking with impact or impact alone in a child younger than 2 years (Choudhary et al., 2018).

## Conclusion

Knowledge regarding AHT has followed the same trajectory as other diseases and conditions in medicine, consequent to the delineation of common clustering of features that are reproducible across time and geography. This consistency is a critical feature of the Bradford Hill criteria for causation (Hill, 2016).

The evolution of understanding of the signs and symptoms of what is called AHT today reflects in the nomenclature: from pachymeningitis hemorrhagica to subdural hemorrhage, from whiplash shaken baby syndrome to AHT.

At the interface between medicine and the law, different approaches to finding the truth and different motives collide. While the sciences strive to get as close to the truth as possible, opposing forces in court seek to prove guilt or cast doubt on it. Alternative explanations are of great importance here, without being subject to scientific scrutiny they would be subjected to in the scientific discourse.

This article has shown that, like other diseases, the core of AHT has been known for centuries, and nuanced knowledge of the scientific underpinning continues to accumulate.

In Part II, we will take the historic and contemporary published literature on AHT and apply the Bradford Hill criteria, identifying similarities and differences with other diseases and conditions.
